# COVID-19 Prognostic Models: A Pro-con Debate for Machine Learning vs. Traditional Statistics

**DOI:** 10.3389/fdgth.2021.637944

**Published:** 2021-12-23

**Authors:** Ahmed Al-Hindawi, Ahmed Abdulaal, Timothy M. Rawson, Saleh A. Alqahtani, Nabeela Mughal, Luke S. P. Moore

**Affiliations:** ^1^Chelsea and Westminster NHS Foundation Trust, London, United Kingdom; ^2^Faculty of Medicine, Imperial College London, London, United Kingdom; ^3^Health Protection Research Unit for Healthcare Associated Infections and Antimicrobial Resistance, Imperial College London, London, United Kingdom; ^4^Centre for Antimicrobial Optimisation, Imperial College London, London, United Kingdom; ^5^King Faisal Specialist Hospital and Research Centre, Riyadh, Saudi Arabia; ^6^Johns Hopkins University, Baltimore, MD, United States; ^7^North West London Pathology, Imperial College Healthcare NHS Trust, London, United Kingdom

**Keywords:** COVID-19, Coronavirus, machine learning, artificial intelligence, linear regression

## Abstract

The SARS-CoV-2 virus, which causes the COVID-19 pandemic, has had an unprecedented impact on healthcare requiring multidisciplinary innovation and novel thinking to minimize impact and improve outcomes. Wide-ranging disciplines have collaborated including diverse clinicians (radiology, microbiology, and critical care), who are working increasingly closely with data-science. This has been leveraged through the democratization of data-science with the increasing availability of easy to access open datasets, tutorials, programming languages, and hardware which makes it significantly easier to create mathematical models. To address the COVID-19 pandemic, such data-science has enabled modeling of the impact of the virus on the population and individuals for diagnostic, prognostic, and epidemiological ends. This has led to two large systematic reviews on this topic that have highlighted the two different ways in which this feat has been attempted: one using classical statistics and the other using more novel machine learning techniques. In this review, we debate the relative strengths and weaknesses of each method toward the specific task of predicting COVID-19 outcomes.

## Introduction

The novel coronavirus SARS-CoV-2 (COVID-19) has placed a significant strain on global healthcare systems. A particular challenge for COVID-19 is the difficulty in predicting individuals who will progress from a viral upper respiratory tract infection to more severe complications (including a dysregulated host response, coinfections, or thrombotic complications). Patients who progress often require critical care and are at significant risk of mortality. With the emergence of potential treatments for both the viral and inflammatory phases of COVID-19, the ability to predict those at high risk and deliver appropriate, prompt therapy could have a significant impact on patient outcomes.

Yet, to help address these critical questions, there is an ever-increasing multimodal pool of “big-data”, with clinical, physiological, radiological, and laboratory parameters to develop, test, and optimize our decision-making pathways. As we consider which input variables may have the greatest influence on patient outcomes, we have a range of techniques, both from classical statistics through to novel artificial intelligence techniques, which we can apply to our clinical questions.

Several prognostic models have already been developed and reported for COVID-19 (1). Many have been developed using traditional statistics, yet machine learning has also been applied to prognostication against a variety of different clinical outcomes (2–4). These machine-learning models bring together statistics and computational programming, with the aim of data analysis without the intrinsic biases inherent in human approaches (Box 1). Before the COVID-19 pandemic, the application of machine learning to infectious diseases had been gaining traction, but to date, very few machine learning programmes are used clinically for prediction and prognostication (5–7). In contrast, prognostic scoring systems developed using traditional statistical methods have been widely implemented in front-line healthcare, including for infectious diseases (8–10). Perhaps foremost among these classical statistics is linear regression, itself a precursor of supervised machine learning, where a model is trained on a set of data with known outcomes with the aim of using what this model learns to predict on data it has not seen before, thus providing clinical insights. What classical statistical methods perhaps lack, however, is flexibility in exploring “unknown” clinical associations, particularly useful in the context of emerging infections, a need that algorithms like neural networks may address.

Box 1 **Data-science tools potentially applicable to COVID-19 prognostication.**

**Table:** 

**Artificial Intelligence (AI)**	**An overarching umbrella term used to denote software that demonstrates intelligence such as learning or problem solving**.
**Machine Learning (ML)**	**A specific subset of artificial intelligence that deals with the creation and validation of models that learn through experience, whether that is supervised (through informing the model of the correct answer during learning) or unsupervised**.
**Neural Network (NN)**	**A common framework that is inspired by the way neurons work in brains. A “neuron” receive inputs from other neurons, sums their outputs adds a bias element (and optionally normalizes the output to given range) and sends its output to another neuron. Useful in supervised tasks where the neuron's inner workings can be tuned to output a specific result given a set of inputs**.
**Deep learning/Deep neural networks (DL/DNN)**	**The finding that layering multiple neurons on-top of each other results in more accurate and precise neural networks**.
**Convolutional neural networks (CNN)**	**A subset of deep neural networks that use the convolutional operator as their basis for learning data features. These have revolutionized working with image and video datasets including the diagnosis of COVID-19 on radiography**.
**Graphics Processing Unit (GPU)**	**Historically used to render graphics in 3D intensive applications like computer games and computer aided design (CAD) where GPUs contain specific matrix multiplication machinery. This matrix multiplication machinery has been repurposed for General Processing on the GPU (GPGPU) leading to quick optimisation of neural networks and very efficient inference**.
**Matrix**	**An array of numbers arranged in a rectangle that can be together a single unit. In the training of ML techniques, these matrices typically represent the weights and biases of each neuron**.
**Tensor**	**A multidimensional array, similar to a multidimensional matrix, where each dimension would represent a different quality of the data. An example is a set of images batched together with a tensor of NxCxWxH where N is the number of images, C is the number of channels in the image, W is the width and H is the height of the image**.

To explore the potential strengths and weaknesses of both traditional statistical methods and machine learning, we present a pro-con debate looking at the current state of the art in these fields, in the context of the wider clinical need for COVID-19 prognostication.

## In Defense of Classical Statistics

### Classical Statistics Are the Foundation of Evidence-Based Medicine

The artificial intelligence (AI) “revolution” in healthcare continues to be promulgated in both scientific and consumer media; yet few, if any, of these innovations have been adopted in day-to-day clinical medicine. Meanwhile, linear regression models like the Acute Physiology and Chronic Health Evaluation (APACHE) score in Intensive Care, CURB-65 score for pneumonia, and the Model of End-Stage Liver Disease (MELD) are in daily clinical use and influence decision making across the globe (8–10). Linear regression models underpin these prognostic scores, acting as the foundation of evidence-based medicine randomized controlled trials. Therefore, before AI techniques are adopted at a large scale into clinical prognostication, we must consider in some detail how they compete with or are perhaps synergistic with, classical statistical techniques.

### Neural Networks Are Opaque and Obfuscate

One of the more recent AI methods to challenge classical statistics has been the resurgence of an approach termed neural networks (Box 1). Neural nets have been investigated for use in clinical medicine since 1976 but suffered a lull due to computational restrictions (11). The recent renewed clinical interest in neural networks has been heralded by the development of convolutional neural networks (CNN) with the concurrent optimisation of matrix multiplication on graphics processing units (GPU), leading to fast training times and faster inference on easy and cheap to acquire hardware. The development of programming frameworks has reduced the barrier of entry for the experimentation in neural networks, leading to the democratization of this technology from what was once a difficult subject (12, 13).

The combination of readily available neural network programming frameworks, large curated clinical datasets, easy-to-learn programming languages, and CNNs have opened a wide window into the regression and classification of highly uncorrelated data, such as clinical radiographic images of computed tomography scans or X-rays (4). While such advances in AI seem potentially attractive, particularly for clinical prognostication, AI systems have been found to have learnt spuriously correlated data, such as a skin cancer classification neural network learning that the presence of a ruler in the image of the lesion accurately classified the presence of melanoma (14, 15).

Neural networks learn exquisite correlations between input variables and the output of interest. It can be argued that the above deficiencies of neural networks are secondary to faults in the dataset, but these faults are very hard to find. While the danger posed by this can be mitigated to some extent by supervision of systems, one might argue that this in some ways defeats the object of AI. Beyond this philosophical argument, in practical terms, supervised systems are difficult to clinically correlate as learnt latent (hidden) variables are difficult to interrogate, difficult to visualize, and impossible to prove coverage of data. In addition, it is currently not mathematically proven that new data entering a system is appropriately represented within the model's internal mechanisms, and reliance on cross-validation is a poor marker of this. It is believed that with sufficient “big data” the neural network may learn an implicit representation of its learning dataset to be sufficiently applied to out-of-sample data, but this is currently impossible to demonstrate, unlike regression models that have closed-form solutions to approximate out of sample performance. This leaves us back at the starting criticism of neural networks, where their hidden mechanics may provide outcomes we as clinicians think useful, but are based upon inputs with no plausible biological relevance.

### Regression Analysis Can Provide Causality and Is Easily Interpretable

In contrast with classical statistical methods, such as multivariable regression, there are decades of research and validation, and when appropriately used can provide robust, simple yet genuine insights into clinical prognosis (8–10). Coefficients in classical multivariate regression have a literal translation, the bigger the coefficients the more important that variable is related to the outcome of interest. Negative coefficients are negatively correlated with the outcome of interest. This allows clinicians to tailor clinical decision-making based on the patient's personal factors making precision, individualized, medicine a reality.

Finding out which variables are related to the outcome of interest from linear regression is inherent in their method, while neural network methods require multiple ablation studies to hint at which variables are correlated to an outcome. Training of linear regression is simple, and ordinary least squares is efficient, fast to train, and is mechanistically transparent. Multilevel, hierarchical, regression models have been successfully trained on tens of thousands of parameters and prior domain knowledge can be inserted into the models using Bayesian techniques (16, 17). Causality (rather than just correlation) can also be demonstrated using classical statistical methods through directed acyclic graphs, a big win if genuine knowledge of the world is required rather than just improved accuracy performance (18).

### Classical Statistics Have Direct Applicability in COVID-19 Prognostication

For COVID-19, many publications have used neural networks to claim unprecedented accuracy for the prediction and classification of COVID-19 outcomes including mortality, ICU admission, and length of stay (2–4, 19). Such an explosion of models has led to the publication of two “living” systematic reviews of those models, the findings of which have been pretty clear: many of those models exhibit “high bias” and are of little clinical use (1, 20). Gupta et al. applied many of the published models to their COVID-19 patient data, highlighting that the best performing, non-biased, model is a simple, well-specified, linear regression composed of age and oxygen saturations alone (20). This makes sense with clinical intuition, where older patients with COVID-19 have higher mortality, and patients who present in worse respiratory failure, as evidenced by lower hemoglobin oxygen saturation (SpO_2_), also have higher mortality. This is not ground-breaking, but it is a transparent finding, which proves that clinical intuition is biologically plausible and is mechanistically probable. While such a simple prognostic model may not add to our understanding, it does perhaps allow us to finesse our pathways and risk stratification more efficiently care for patients when our healthcare services are at near-maximal capacity.

### The Machine Learning Revolution Is Inevitable

#### Why Is Machine Learning So Powerful?

Consider a computerized tomography (CT) scan of the chest for a patient with COVID-19. The principal finding will be atypical or organizing pneumonia in up to 97% of patients with a severe infection (21–24). However, the images produced by the CT scanner are large, highly dimensional images, and therefore the data within them must be highly structured in some way so as to represent organizing pneumonia, and not random noise, or indeed a picture of something else.

The manifold hypothesis aims to explain this phenomenon. It posits that natural data lies on a low-dimensional manifold within the high-dimensional space where it is encoded (25). In other words, data pertaining to a particular class (for example, CT images of the chest) are a highly structured subset of all possible inputs for that class (i.e., all possible images/pixel values which can exist in the same size of image). This means that machine learning algorithms only need to learn a few key features from the data to be effective. This is analogous to physicians carefully picking a few important variables in multivariable regression analysis to answer a particular research question. The key difference is that the best possible features from any given highly dimensional dataset may turn out to be complicated functions of the original variables. The function of machine learning algorithms is to find these complex key features within a forest of data, which is a task that is not possible with classical statistical techniques.

#### Minimizing Bias While Maximizing Data Utilization

Bias, defined as a feature of a statistical technique or of its results whereby the expected value of the results differs from the true underlying quantitative parameter being estimated, is of paramount importance during all phases of model development, including training and validation. Christodolou et al. conducted a metaregression analysis that failed to demonstrate the improved discriminative performance of machine learning algorithms over logistic regression for clinical prediction models (6). While the area under the receiver operating curve (AUC) was on average no different between the two techniques when comparisons had a low risk of bias, machine-learning algorithms had improved performance among studies where there was a higher risk of bias, a potential advantage of machine learning algorithms over human-led statistical analysis. However, the systematic review was unable to report on measures of calibration due to poor reporting of this metric in the studies considered. There is a clear need therefore that future machine learning prognostic studies report calibration metrics and include a full report of all modeling steps, with particular adherence to the TRIPOD guidelines (26).

Predictive models in healthcare that utilize large datasets and a large number of parameters have demonstrated improved performance with machine-learning algorithms. A predictive model designed to forecast the development of acute kidney injury (AKI) analyzed data from 703,782 adults across 172 inpatient and 1,062 outpatient sites and considered 3,599 clinically relevant features that were provided to the baseline at each step (27). In all stages of AKI, classical logistic regression yielded lower precision-recall and receiver operator areas under the curve (PR AUC and ROC AUC, respectively) than Random Forest and Gradient Boosted Trees, which themselves yielded lower PR AUCs and ROC AUCs than deep learning approaches, such as intersection recurrent neural networks and long-short-term-memory networks (27).

In a systematic review and critical appraisal of current predictive models for COVID-19, Wynants et al. noted that all the 145 predictive models considered were at some risk of bias for a variety of reasons, ranging from lack of accounting for censoring (leading to selection bias), to using small sample sizes and subjective variables, and not reporting on calibration measures. They echo the importance of using the TRIPOD guidelines in future predictive work (1). When using the TRIPOD guidelines to develop statistical and machine learning predictive models for COVID-19 prognosis, including the use of Cox regression analysis to account for censoring, reporting the validation, discrimination, and calibration of both techniques; and comparing both model ROC AUCs on the same dataset, there is evidence that machine-learning techniques outperform classical methods, even in moderately sized datasets (19).

#### Machine Learning; Not Quite as Opaque as Initially Thought

A key advantage frequently attributed to classical regression analysis is that each variable in the regression is assigned a coefficient by the model. The direction and magnitude of this coefficient directly relate to the direction and magnitude of the association between the variable considered and the outcome investigated. In contrast, the renewed interest in neural networks has been met with a steady criticism that such networks are non-transparent and that their predictions are not traceable by humans due to their multilayer, non-linear structure (28). However, explainable deep learning has recently become an active area of intense research which has produced three principled branches of explanatory methods, each with two subdivisions. Namely, visualization methods through perturbation or back-propagation, distillation methods through model translation or local approximation, and intrinsic techniques such as the use of attention mechanisms or joint training (29, 30).

Lundberg et al. utilized Shapley additive explanation, which is a variant of explanation through back-propagation work proposed by Shrikumar et al. which predicts near-term risk of hypoxaemia during anesthesia care, whilst explaining the patient- and surgery-specific factors leading to that risk in real-time (31, 32). Indeed, this technique can be applied to arbitrarily complex network architectures and has been used with success in deep learning prognostic models for COVID-19 to highlight salient patient characteristics leading to individual mortality predictions (2).

#### Optimizing Workflow Is Essential With Clinical “Big-Data”

Machine-learning algorithms can be easily implemented into end-to-end programmes capable of taking any desired data type as their input and producing relevant results (e.g., by scanning a dermatologic image through a phone app to produce a prediction of whether a skin lesion is malignant). While the important hazards of using inaccurate or potentially biased data cannot be overstated, such systems have nonetheless been able to outperform panels of expert specialists (33).

Furthermore, machine-learning algorithms can be used to predict multiple endpoints from a single feature set, which is difficult with classical statistical analysis. For example, Hofer et al. developed and validated a neural network from 59,981 surgical procedures capable of predicting postoperative mortality, AKI, and reintubation from a single feature set (34). Their model achieved a greater ROC AUC for their outcomes than the well-established ASA physical status score alone. This feature is particularly applicable to COVID-19, where predictive models need to be able to respond to changing management paradigms, changing outcomes, and evolving diseases complications.

## Conclusion

**Figure 1:**
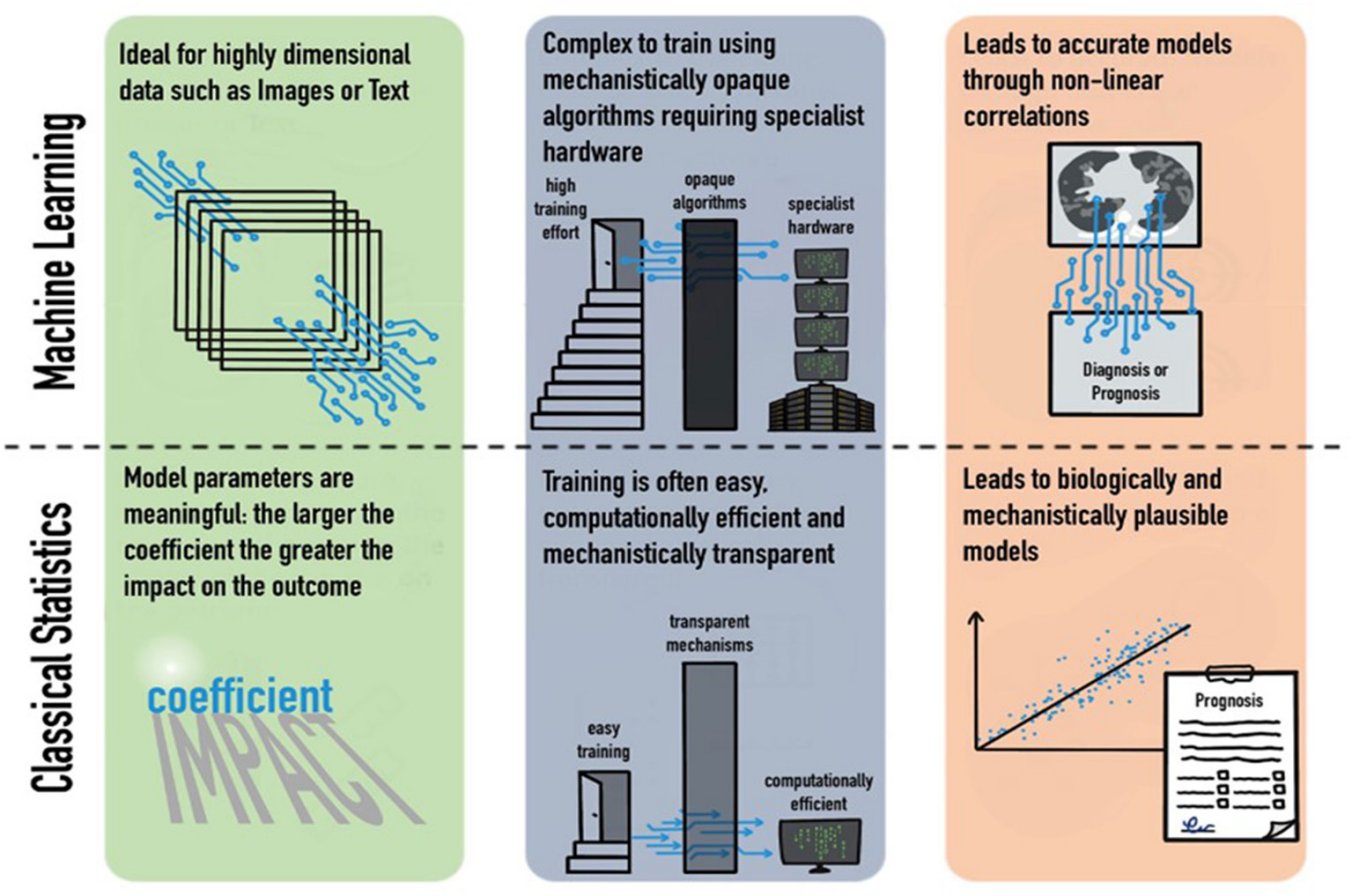
Strengths and weaknesses of machine learning and classical statistics in their domains, training requirements, and outputs.

There is little doubt that our ability to collect increasingly multimodal, highly dimensional clinical data will increase dramatically in the next few years, as typified by the formation and mandate of government bodies such as the United Kingdom's NHSX unit. Machine-learning techniques can produce models which are capable of utilizing a large array of multimodal data to produce multiple predictions simultaneously. This has been demonstrated by its promising use in the COVID-19 pandemic to produce ever more accurate predictions. However, the application of these complex models does not obviate the need for classical statistical analysis; causality and biological mechanistic plausibility remain in the realm of classical statistics (Figure 1; Table 1). Each technique has its merits, and blind application of either method has significant scientific ramifications.

**Table 1:** Uses and strengths of classical statistics vs. machine learning in COVID-19 prognostic modeling.

**Classical statistics**	**Machine learning**
In active clinical use	Useful for non-correlated data, like text or images
Foundation of modern medicine	Increasing in use across all areas of medicine
Mechanistically transparent	Requires little data pre-engineering
Easy to interrogate and can provide causality	Explainability is an active area of research with SHAP values explaining per patient predictions
Best non-biased estimator of mortality for COVID-19	Provided state of the art prognostic models across many domains

## Author Contributions

AA-H and LM: conceptualization. AA-H, AA, TR, and LM: writing—original draft. AA-H, AA, TR, SA, NM, and LM: writing—review and editing. All the authors have read and approved this manuscript and agree as to its contents.

## Funding

This work was funded by the Chelsea Infectious Diseases Research (CINDER) Fund at CW+ Charity.

## Author Disclaimer

The views expressed in this publication are those of the authors and not necessarily those of the NHS, the National Institute for Health Research, or the UK Department of Health.

## Conflict of Interest

All authors have completed ICMJE forms for Disclosure of Potential Conflicts of Interest and declare the following: NM has received speaker fees from Beyer (2016) and Pfizer (2019–2021) and received educational support from Eumedica (2016) and Baxter (2017). LM has consulted for/received speaker fees from DNAelectronics, (2015–18), Dairy Crest (2017–2018), Profile Pharma (2018–2019), bioMerieux (2013–2021), Eumedica (2016–2021), Umovis Lab (2020), Pfizer (2018–2021), Shionogi (2021), Pulmocide (2021), Sumitovant (2021), and Kent Pharma (2021) and received research grants from the National Institute for Health Research (2013–2019), Leo Pharma (2016), CW+ Charity (2018–2021), and LifeArc (2020–2021).

## Publisher's Note

All claims expressed in this article are solely those of the authors and do not necessarily represent those of their affiliated organizations, or those of the publisher, the editors and the reviewers. Any product that may be evaluated in this article, or claim that may be made by its manufacturer, is not guaranteed or endorsed by the publisher.
